# The effect of Rho kinase inhibition on long-term keratinocyte proliferation is rapid and conditional

**DOI:** 10.1186/scrt449

**Published:** 2014-04-28

**Authors:** Sandra Chapman, David H McDermott, Kui Shen, Moon Kyoo Jang, Alison A McBride

**Affiliations:** 1 Laboratory of Viral Diseases, National Institute of Allergy and Infectious Diseases, National Institutes of Health, Bethesda, MD 20892, USA; 2 Laboratory of Molecular Immunology, National Institute of Allergy and Infectious Diseases, National Institutes of Health, Bethesda, MD, USA; 3 Bioinformatics and Computational Biosciences Branch, National Institute of Allergy and Infectious Diseases, National Institute of Allergy and Infectious Diseases, National Institutes of Health, Bethesda, MD, USA

## Abstract

**Introduction:**

We previously demonstrated that the lifespan of primary human keratinocytes could be extended indefinitely by culture in the presence of the Rho kinase (ROCK) inhibitor Y-27632. This technique has proven to be very useful in diverse areas of basic and clinical research.

**Methods:**

In this follow-up study we determine whether the continual presence of Y-27632 is required for sustained proliferation. We also test whether different ROCK inhibitors can be used for this technique and whether it can also promote indefinite proliferation of animal keratinocytes. We measure keratinocyte gene expression, proliferation, behaviour and lifespan in the presence and absence of Y-27632.

**Results:**

We demonstrate that the extension of lifespan observed by culture of keratinocytes in the presence of fibroblast feeders and a ROCK inhibitor is reversible and that cells senesce gradually when the inhibitor is removed from the medium. Conversely, keratinocytes that are close to the end of their replicative life span can be revived by ROCK inhibition. We demonstrate that different inhibitors of ROCK can also efficiently extend the lifespan of human keratinocytes and that ROCK inhibition extends the lifespan of animal keratinocytes derived from mouse and bovine epithelia. Gene expression analysis of human epidermal keratinocytes cells grown in the presence of Y-27632 demonstrates that ROCK inhibition primarily inhibits keratinocyte differentiation. Live-imaging of keratinocytes cultured with ROCK inhibitors show that the effect of ROCK inhibition on cellular proliferation is immediate and ROCK inhibited cells proliferate rapidly without differentiation or stratification.

**Conclusions:**

ROCK inhibition rapidly and conditionally induces indefinite proliferation of keratinocytes. This method has far-reaching applications for basic research, as well as for regenerative and personalized medicine.

## Introduction

Primary keratinocytes have a finite lifespan in culture, but we previously demonstrated that culture in the presence of a Rho kinase (ROCK) inhibitor greatly increased the proliferation and resulted in apparent immortalization of human keratinocytes derived from several anatomical sites [1]. We demonstrated that the resulting keratinocytes were very similar to primary keratinocytes in that they had a normal karyotype, an intact DNA damage response and could differentiate into a stratified epithelium [1]. These cells have proven to be very useful for basic research studies and for clinical research. For example, it has allowed keratinocytes with specific properties, such as the ability to be efficiently transfected, to be isolated and used for a wide range of experiments [2]. This technique has also enabled the efficient procurement and culture of keratinocytes from biopsies of patients with infectious, genetic and malignant diseases [3-5]. Furthermore, the lifespan of non-keratinocyte epithelial cells derived from normal or cancerous tissue can be extended indefinitely by culture with ROCK inhibitors [3]. Others have found that this method enhances lentiviral transduction of keratinocytes and enhances the development of human skin equivalents (as long as the ROCK inhibitor is removed during differentiation) [6]. Thus, this culture method also has enormous promise for gene therapy.

In this follow-up study, we show that this indefinite extension of lifespan is conditional and after removal of the ROCK inhibitor, cells slow in growth and senesce after a few passages. We also show that the ROCK inhibitor can be added at late stages of the replicative life span, when cells are close to senescence, and it will still efficiently promote indefinite proliferation of the cells. We also extend these studies to show that animal keratinocytes can be induced to proliferate indefinitely using this technique. We show that several other inhibitors of the Rho kinase can also induce indefinite proliferation of keratinocytes. Using gene expression analysis, we show that one of the primary results of ROCK inhibition is inhibition of differentiation. Furthermore, this effect is immediate and increased proliferation can be observed within days of addition of the ROCK inhibitor.

## Methods

### Cells

Neonatal human keratinocytes were isolated from human foreskins, which were collected with informed consent of parents or guardians and with approval from the Institutional Review Boards at NIH in adherence to the Declaration of Helsinki Principles. Adult human keratinocytes were collected from a small punch biopsy from the inner arm that was collected after subjects signed informed consent consistent with the Declaration of Helsinki under the appropriate clinical protocol with approval from the NIAID Institutional Review Board at NIH. Keratinocytes were isolated from tissues as described previously [1]. Bovine keratinocytes were harvested from a third trimester foetal calf provided by Pel-freez Biologicals (Rogers, Arkansas, USA). Newborn C57Bl/6NCr mouse keratinocytes were a gift from Wendy Weinberg, Food and Drug Administration. All animal work was performed in accordance with NIH (National Institutes of Health) established guidelines and accepted standards of humane animal care under protocols approved by the Animal Care and Use Committee of the Center for Biologics Evaluation and Research of the Food and Drug Administration.

### Cell culture

Keratinocytes were cultured in F-medium (3:1 (v/v) F-12-DMEM, 5% foetal bovine serum (FBS), 0.4 μg/ml hydrocortisone, 5 μg/ml insulin, 8.4 ng/ml cholera toxin, 10 ng/ml epidermal growth factor (EGF), 24 μg/ml adenine, 100 U/ml penicillin, 100 μg/ml streptomycin) in the presence of irradiated J2 3T3 feeder cells [7]. Mouse keratinocytes were cultured both in F-medium and in low calcium DMEM supplemented with 10% chelated FBS, as described previously [8], but in both cases they were grown in the presence of irradiated J2 3T3 cells. Cells were subcultured when they were approximately 80 to 90% confluent by removing the fibroblast feeder cells with Versene (Life Technologies, Grand Island, NY, USA), counting the keratinocytes and passing between 1 to 2 × 10^5^ cells to a new 10 cm plate of irradiated J2 3T3 feeder cells. Population doubling was calculated as: PD = 3.32 (log (# cells harvested/# cells seeded)). Cells were grown in the presence or absence of the ROCK inhibitors, as indicated. Senescence was defined as growth rate (population doubling/day) ≤0.2 within the time period of one month.

### Inhibitors

The ROCK inhibitors Y-27632, fasudil hydrochloride, HA1000 hydrochloride and GSK 429286 were obtained from Tocris Biochemicals, Bristol, United Kingdom.

### Microarray analysis

RNA was prepared using an RNeasy Mini RNA purification kit (Qiagen Science Inc., Gaithersburg, MD, USA) from adult keratinocytes isolated from cutaneous skin and cultured for either three or four passes (two replicates: two different passes of cells from one individual) in the presence or absence of 10 μM Y-27632. Total RNA was purified, and analysed for integrity using the Agilent RNA 6000 nano kit on a 2100 Bioanalyzer (Agilent Technologies, Inc., Santa Clara, CA, USA). cDNA was prepared using the Illumina® TotalPrep™ RNA Amplification Kit (Life Technologies) and gene expression was analysed by hybridisation to Illumina whole genome Human HT-12 v4.0 BeadChip. Gene ontology analysis was conducted using Gene Ontology enRIchment anaLysis and visuaLizAtion (GORILLA) [9]. Microarray data have been uploaded to Gene Expression Omnibus (GEO) under accession number GSE52515). Genes that were significantly up- or down-regulated (*P* <0.05) are listed in Additional file 1: Table S1. To further analyse differential gene expression, the microarray data were preprocessed by quantile normalisation and probes with low expression values and small variations across samples were filtered out. Differentially expressed genes were identified using the R software package Limma [10] and are presented as a heatmap with hierarchical clustering. The list of differentially expressed genes is listed in Additional file 1: Table S1.

### Time lapse live cell imaging

Irradiated J2-3 T3 feeders and human foreskin keratinocytes were plated in six-well culture dishes in F-medium with or without Y-27632 and were imaged for up to six days by Phase Contrast using an Incucyte incubator microscope (Essen Bioscience, Ann Arbor, MI, USA). Confluence was calculated using the Incucyte Zoom software.

## Results

### The proliferative effect of Y-27632 on human keratinocytes is conditional

We showed previously that the lifespan of human keratinocytes can be extended indefinitely by culture in the constant presence of the ROCK inhibitor Y-27632 [1]. We also showed that Y-27632 inhibited keratinocyte stratification and differentiation in an organotypic skin equivalent, but that removal of Y-27632 from the culture medium allowed the cells to differentiate normally [1]. This suggested that ROCK inhibition was conditional and reversible; however, the conditional nature of ROCK inhibition on long term keratinocyte proliferation has not been examined. Thus, we carried out a series of experiments to determine whether continual ROCK inhibition was required for sustained proliferation.

**Figure 1:**
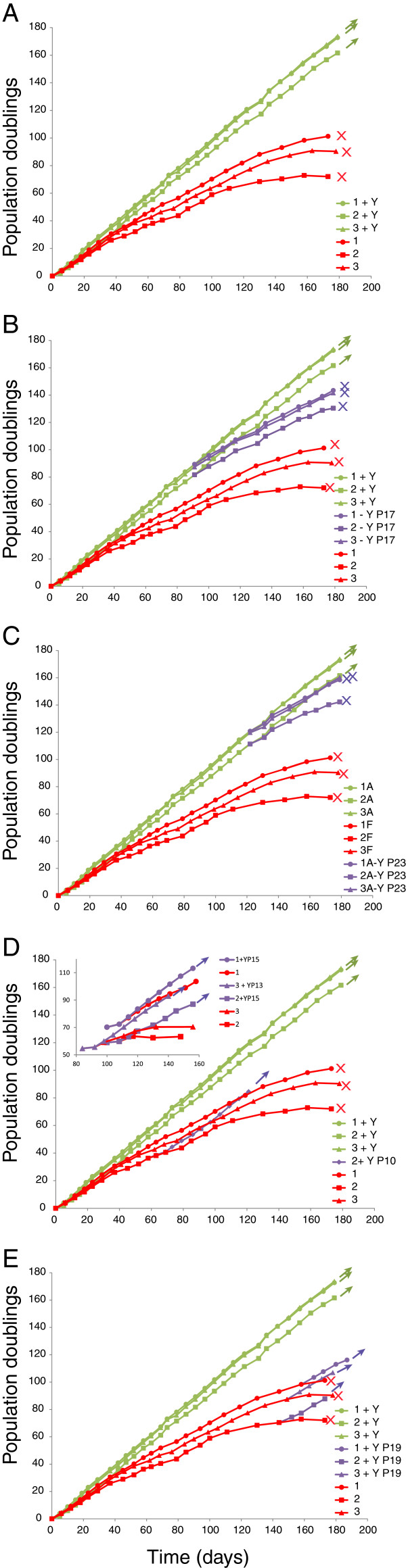
**Addition and removal of Y-27632 during long-term culture. A**. Growth rate of three strains of foreskin keratinocytes from different donors cultured for at least 180 days in the presence or absence of 10 μM Y-27632. Cells cultured with Y-27632 continued to proliferate logarithmically (arrows) while untreated cells senesced at the times indicated by X. **B**. Y-27632 was removed from the three strains of keratinocytes cultured in its presence at pass 17. The subsequent growth curves are indicated in purple and senescence is indicated by X. **C**. Y-27632 was removed from the three strains of keratinocytes cultured in its presence at pass 23. The subsequent growth curves are indicated in purple and senescence is indicated by X. **D**. Y-27632 was added to keratinocyte strain 2 at pass 10. The resulting growth curve is shown in purple. In the inset, all three strains were cultured after cryogenic storage with Y-27632 added at pass 13 or 15 (as indicated). The resulting growth curves are shown. **E**. Y-27632 was added to keratinocyte strains at pass 19. The resulting growth curves are shown in purple.

In these experiments we removed Y-27632 at different times from cells that had been continually grown in its presence, and added it to cells that were only a few passages from senescence. The controls for these experiments are shown in Figure 1A: human foreskin keratinocytes (HFKs) from three different donors were cultured in parallel in the presence or absence of Y-27632. Cells were passed at 80 to 90% confluence and untreated cells were passed until senescence (indicated by X). As shown previously, in the absence of Y-27632 all three strains slowed in growth and reached senescence by population doubling 72 (strain 2), 90 (strain 3) and 101 (strain 1). However, in the presence of 10 μM Y-27632, cells from all three strains had undergone approximately 160 to 170 population doublings in the same time period and continued to grow logarithmically. It is noteworthy that each strain of keratinocytes had an inherent growth rate. For example, keratinocyte strain #2 consistently had a slower growth rate than the other strains, both in the presence or absence of Y-27632, and it senesced earlier than the other two strains.

In the representative experiments shown in Figure 1B,C, Y-27632 was removed from the culture medium from all three strains of keratinocytes at two different passes (P17 and P23). Note that the controls from panel 1A are reproduced in panels B and C as a reference. In both cases, and for all three strains, the growth rate of the cells slowed and after several passages the cells senesced. We have reproduced this observation multiple times with different cell strains and only once or twice did a rare keratinocyte colony continue to proliferate after removal of Y-27632. We presume that these rare cases of observed spontaneous immortalization are due to rare genetic alterations acquired after long-term culture (for example, [11]). Notably, the number of passes remaining until senescence after removal of Y-27632 is dependent on how many passes the cells have been cultured in the inhibitor: cells that had been grown longer in Y-27632 senesced more quickly, indicating that they retained an internal replicative clock.

Conversely, Y-27632 was added to keratinocytes that had been cultured for 10, 13, 15 (Figure 1D) or 19 (Figure 1E) passes in the absence of the ROCK inhibitor. Again, note that the controls from panel 1A are reproduced in panels D and E as a reference. Remarkably, the rate of proliferation increased immediately, even in the late pass cells that were only a few cell divisions from senescence. This indicates that ROCK inhibition is not selecting for a rare cell population in freshly isolated keratinocytes, and is able to induce proliferation even in keratinocytes that have only a few replicative cycles remaining.

### ROCK inhibition induces animal keratinocytes to proliferate indefinitely

**Figure 2:**
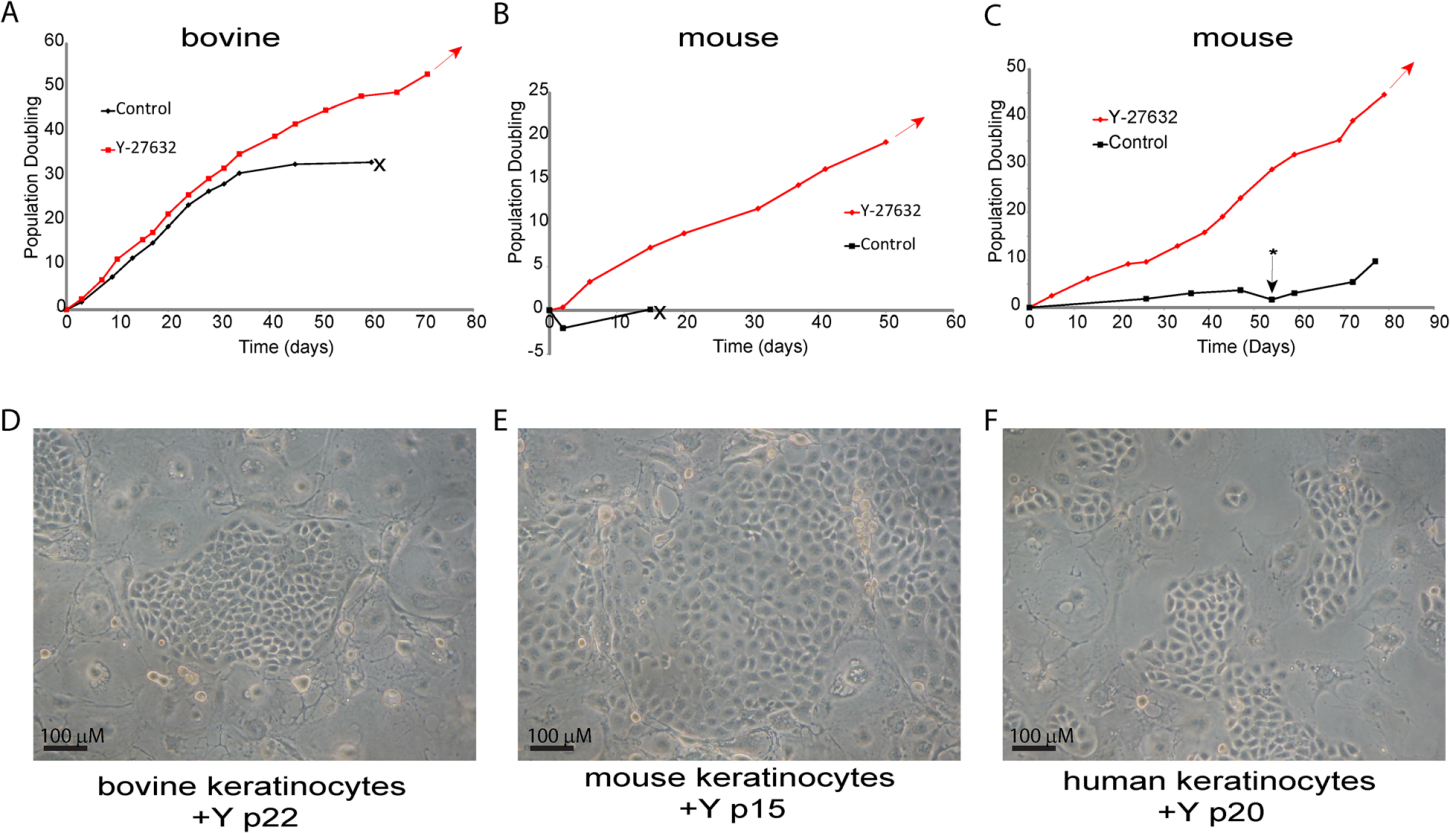
**Animal keratinocytes proliferate indefinitely in the presence of Y-27632.** Growth curves of foetal bovine keratinocytes **(A)** or new-born mouse keratinocytes **(B and C)** grown in the absence or presence of Y-27632. Cells in **A** and **C** were grown in F-medium in the presence of J2 3T3 feeders. Cells in **B** were grown in low calcium DMEM in the presence of J2 3T3 feeders. The arrow represents continued proliferation and the X represents senescence. In panel **C**, the *symbol represents a stage when most cells were senescent; however, a few very rare cells spontaneously transformed and began to proliferate. Panels **D**, **E** and **F** show phase contrast images of bovine **(D)**, mouse **(E)**, and human keratinocytes cultured for the passes shown in the presence of Y-27632. DMEM, Dulbecco’s modified Eagle’s medium.

To date, most studies have examined the effect of ROCK inhibition on human keratinocytes, although our colleagues have shown that Y-27632 effectively induces proliferation of canine keratinocytes [3]. We also show here that foetal bovine keratinocytes and new born mouse keratinocytes can be induced to proliferate and bypass senescence when grown in co-culture with irradiated J2 3T3 fibroblasts in Rheinwald-Green F-medium supplemented with 10 μM Y-27632. Figure 2 shows growth curves of bovine and mouse keratinocytes cultured in the absence or presence of Y-27632 and images of the resulting keratinocytes. The mouse keratinocytes were cultured either in F-medium in the presence of J2 3T3 feeders (Panel 2C) using the same conditions as for human keratinocytes or were grown in low calcium DMEM (Panel B), although also in the presence of J2 3T3 feeders, which is non-traditional for this culture method [12]. Although we have reproduced the enhanced proliferation of mouse keratinocytes several times, these data should be taken as preliminary but very promising, as we have not carried out extensive growth curves or analysis of the treated keratinocytes. However, in agreement, Bilousova and Roop also report that Y-27632 prolongs the survival of normal mouse keratinocytes in culture [13].

### Effect of different ROCK inhibitors on keratinocyte immortalization

**Figure 3:**
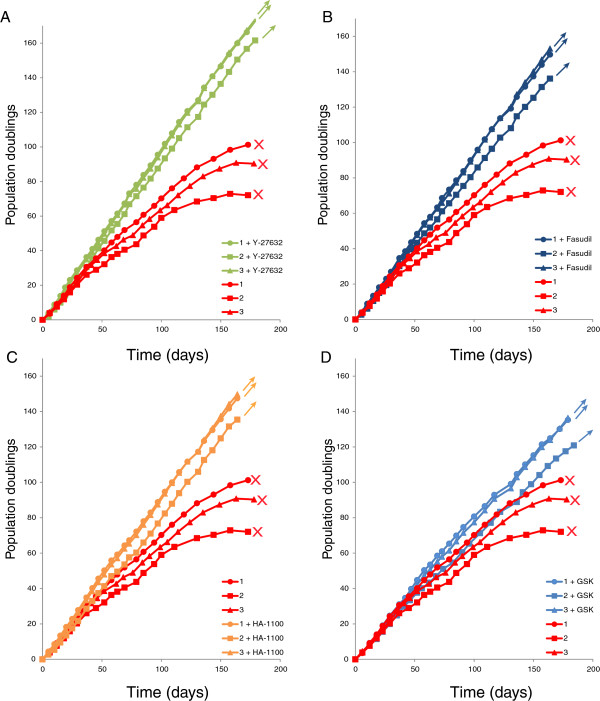
**Effect of different ROCK inhibitors on keratinocyte immortalization. A**. Growth curves of the three strains of foreskin keratinocytes cultured in the presence or absence of 10 μM Y-27632, and shown in Figure 1, are reproduced here for comparison with panels **B**, **C** and **D**. **B**, **C**, **D**. Growth curves of the same three strains of foreskin keratinocytes shown in Figures 1 and 2A were cultured in the absence (red) or presence of 20 μM fasudil (**B**; dark blue line), 20 μM HA-1100 (**C**, orange line) or 100 nM GSK 429286. (**D**; light blue line).

Most studies on the role of Rho-associated kinases on keratinocyte proliferation have used Y-27632 to inhibit ROCK [1,6,14]. Y-27632 is a potent inhibitor of ROCK 1 and 2, but it also has some inhibitory effects on PKC, cAMP-dependent protein kinase and myosin light-chain kinase [15]. Therefore, we tested additional ROCK inhibitors to determine whether they could also promote keratinocyte proliferation. Fasudil hydrochloride (HA-1077) is a well-known vasodilator [16] that inhibits ROCK 1 and 2 as well as cAMP-dependent protein kinase. HA1000 hydrochloride is a hydroxylated metabolite of HA-1077 and is 100 times more selective for ROCK 1 and 2 than for other kinases. Finally, GSK 429286 is a potent ROCK1/2 inhibitor that is active at much lower concentrations (IC50 14 nM) [17]. In parallel, cells were treated with 10 μM Y-27632, 20 μM fasudil hydrochloride, 20 μM HA1000 hydrochloride, or 100 nM GSK 429286. Cells were passed for the times and population doublings shown in Figure 3, by which time it was clear that treatment with all four ROCK inhibitors had stimulated the keratinocytes to bypass senescence and in each case the resulting cells were still highly proliferative.

### Y-27632 inhibits keratinocyte differentiation and stimulates cell division and nucleic acid metabolism

**Figure 4:**
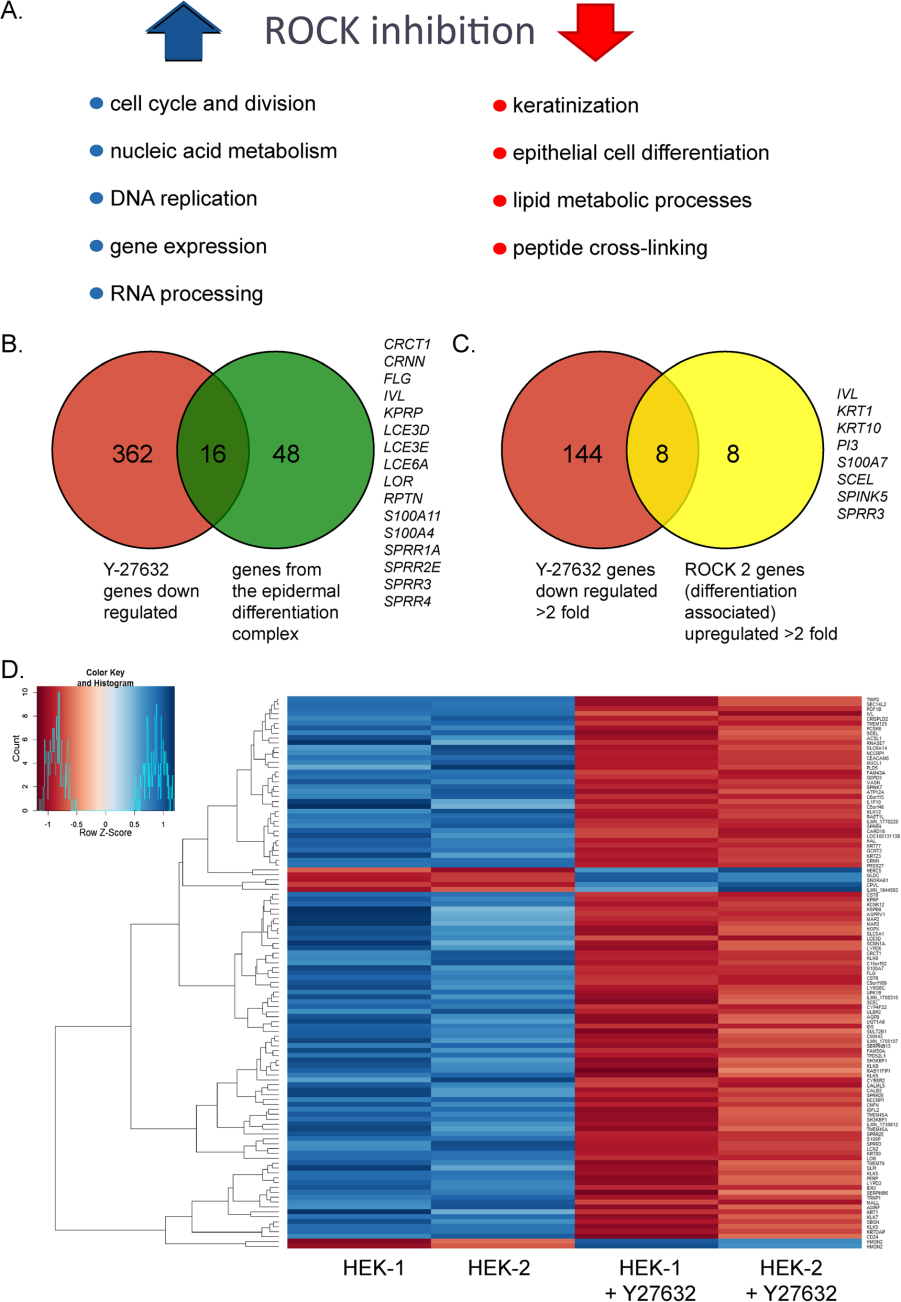
**Gene expression analysis of human epidermal keratinocytes cultured in the presence or absence of Y-27632.** Adult human keratinocytes were cultured for several passes in the presence or absence of 10 μM Y-27632. RNA was isolated at passage 3 and passage 4 and was analysed for gene expression by microarray analysis. **A**. The major gene ontology (GO) categories up-regulated (blue arrow) and down-regulated (red arrow) are shown. Additional file 2: Table S2 shows the complete list of statistically significantly changed GO categories. **B**. A comparison of significantly (*P* <0.05) down-regulated genes from this study with genes encoded by the epidermal differentiation complex [18]. The genes common to both categories are listed to the right. **C**. A comparison of genes from this study down-regulated at least two-fold by Y-27632 with genes up-regulated by ROCK 2 and associated with keratinocyte differentiation as described by McMullan *et al*. [19]. The genes common to both categories are listed to the right. **D**. The microarray data were further analysed for differential gene expression using the R software package Limma [10] and are presented as a heatmap with hierarchical clustering. Down-regulated genes are shown in orange and up-regulated in blue. The list of differentially expressed genes is listed in Additional file 1: Table S1.

To further study the role of Y-27632 on human keratinocyte proliferation we measured gene expression in cells cultured in the absence or presence of the Y-27632. To our knowledge, global gene expression has not been examined in primary human keratinocytes cultured in the presence or absence of Y-27632 (GEO). Cutaneous adult keratinocytes were cultured for either three or four passes after procurement in 10 μM Y-27632 and RNA was isolated from each pass and analysed by Illumina whole genome microarrays. The log 2 ratio of the difference in the level of expression of each gene was calculated (between the means of the treatment groups ((cells cultured in the presence of Y-27632 - level of expression in cells cultured in the absence of Y-27632)). A *P*-value was calculated for each difference and further adjusted for multiple testing (using the False Discovery Rate statistic). The genes were sorted in rank order of the mean of the difference in expression level and submitted to GORILLA (Gene Ontology enRIchment anaLysis and visuaLizAtion tool) [9]. This showed that treatment with the ROCK inhibitor dramatically down-regulated genes involved in keratinocyte differentiation. For example, loricrin and filaggrin were radically down-regulated by Y-27632 treatment. This is very consistent with our previous study showing that Y-27632 inhibited differentiation of keratinocytes and stratification in an organotypic raft model [1]. In contrast, genes involved in cell division and nucleic acid biosynthesis were up-regulated, consistent with rapid proliferation. Figure 4A shows an overview of these data, and gene ontology (GO) terms with *P*-values <10^−9^ are listed in Additional file 2: Table S2. Additional file 1: Table S1A and 1B lists all genes that were significantly (*P* <0.05) up-regulated or down-regulated by culture in Y-27632 and the complete dataset is available at GEO: GSE52515.

To further emphasize that many Y-27632 down-regulated genes are associated with keratinocyte differentiation, we compared the significantly (*P* <0.05) down-regulated gene list with genes from the epidermal differentiation complex (EDC), a genomic locus encoding over 60 keratinocyte differentiation genes [18]. As shown in Figure 4B, 25% of EDC genes are down-regulated after culture in Y-27632. McMullan and colleagues have also previously proposed that keratinocyte differentiation is regulated by the ROCK signalling pathway [19]. Consistent with our findings, they showed by gene expression analysis that ROCK2 induced genes were associated with keratinocyte differentiation. Figure 4C shows that 50% of differentiation-associated genes up-regulated at least two-fold by ROCK2 [19] were also down-regulated at least two-fold by treatment with Y-27632.

Genes that were differentially expressed in the presence of Y-27632 were further identified using the R software package Limma [10] and the results are presented as a hierarchical heatmap in Figure 4D. As can be seen, the majority of differentially expressed genes are down-regulated after Y-27632 treatment. The list of differentially expressed genes is listed in Additional file 2: Table S2.

Additional file 1: Table S1 also contains a dataset of expression of genes previously implicated in keratinocyte proliferation and stem cell identity. There is an 89% and 24% increase in expression of TAp63alpha and ΔNp63alpha, respectively, after culture in Y-27632. CD71 is also increased by over 70% after culture with the ROCK inhibitor and both of these markers are characteristic of proliferating cells. Conversely, ROCK inhibition results in a 20% and 45% decrease in expression of NOTCH 2/3 factors that can promote differentiation. However, the observed changes are minimal and not statistically significant (further hindered by our small sample size of two replicates). Overall, and as discussed below, there are no major changes in the level of factors previously implicated in epidermal stem cell identity at the transcriptional level.

### Keratinocytes begin to rapidly proliferate upon exposure to Y-27632

**Figure 5:**
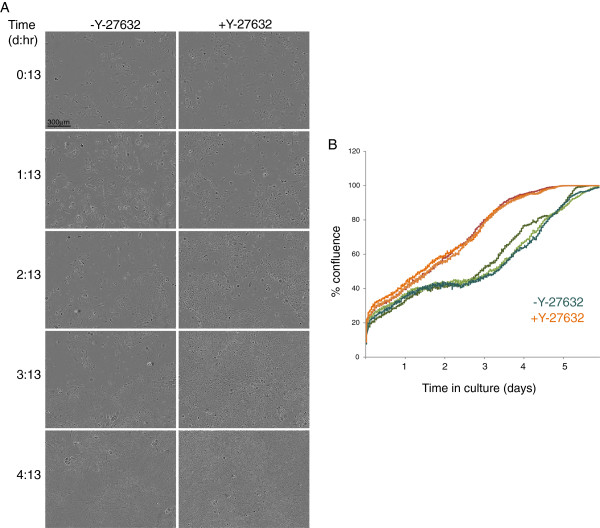
**Live cell imaging of adult human keratinocytes cultured in the presence or absence of Y-27632. A**. Human foreskin keratinocytes at passage 6 were plated in triplicate in the presence or absence of Y-27632. Still images from live movies of each culture condition are shown at the times indicated to represent the confluence of the colonies. The corresponding movies are available as Supplementary data. **B**. Growth curves were calculated using time-lapse photography to measure confluence of cultures for up to six days (by Incucyte ZOOM software).

The long-term effects of Y-27632 on keratinocyte proliferation could be due to a continual selection for a small subset of proliferative, stem-like cells or it could be due to a conversion of the entire culture population to a more proliferative state. To gain more insight into the short-term effects of Y-27632, we plated early-pass foreskin keratinocytes in the presence or absence of Y-27632 and observed their behaviour by time-lapse photography using an Incucyte incubator microscope. Keratinocytes plated for the first time in Y-27632 were observed for up to six days. The proliferative effect of Y-27632 can be observed as early as one to two days after plating and appears to affect a large proportion of cells in the population. In the Rheinwald-Green culture system the keratinocytes migrate and cluster together in small colonies that expand to fill the culture dish. In the absence of Y-27632, the cells in the centre of the colonies appear to enlarge and differentiate, as the colonies grow larger. However, in the presence of Y-27632 even very large colonies are completely packed with dividing, proliferative cells. The keratinocytes pack tightly into the monolayer and growth does not appear to be inhibited when cells contact each other. The cells only cease to multiply when the plate is completely confluent and the cells are packed into the cell culture dish as tightly as is physically possible. However, even then they do not pile up on each other, or differentiate. This is very consistent with the microarray data and shows that the cells are highly proliferative, but resistant to differentiation and stratification. Figure 5A,B shows images and growth curves derived from a six-day time course and a representative movie is shown in Additional file 3: Movie S1. These results also show that the effect of Y-27632 on keratinocyte proliferation is observed within one to two days of the addition to the culture and is not due to selection of a highly proliferative minority of cells.

## Discussion

We have previously shown that the addition of the Y-27632 ROCK inhibitor to the Rheinwald-Green co-culture method greatly improved the proliferative capacity of primary human keratinocytes [1]. In this report, we further characterize this observation and show that increased proliferation and extension of lifespan is conditional and reversible; when the ROCK inhibitor is removed, keratinocytes senesce within a few passes. Cells appear to retain an internal clock, as cells that were cultured for longer periods in Y-27632 senesce faster than those that were only cultured for a shorter period with the ROCK inhibitor. Cells close to senescence before the addition of Y-27632 also senesce rapidly upon its removal. Technically, this is very useful as cryopreserved cells that are capable of only a few remaining cell divisions can be induced to proliferate with Y-27632.

We have previously shown that telomerase levels are up-regulated in keratinocytes cultured with Y-27632 [1] but this is most likely due to co-culture with fibroblast feeder cells [20]. In support of this, our gene expression analysis shows only a minimal (10%) increase in hTERT expression when Y-27632 is added to the keratinocyte feeder co-culture (Additional file 1: Table S1 Sheet D). We showed previously that, despite high telomerase expression, the telomeres erode with passage but finally stabilize at a defined length [1]. We speculate that the time to senescence after removal of Y-27632 is determined by the remaining length of the telomeres. The ability of cells to continue to grow for several passages after removal of the ROCK inhibitor has great utility as this allows experiments to be conducted on healthy, proliferative cells in the absence of pharmaceutical inhibition. It also further emphasizes our original observation that these cells, while conditionally immortalized, retain the properties of primary keratinocytes [1].

ROCK inhibitors interfere with the ability of keratinocytes to differentiate, indicating that this pathway is essential for differentiation [1,19,21]. We show here by gene expression analysis of primary human epidermal keratinocytes cultured with Y-27632 for three or four passages that the most dramatic change in gene expression is down-regulation of many genes expressed during keratinocyte differentiation. There are two ROCK kinases (1 and 2) that are downstream effectors of the Rho GTPase pathway in keratinocytes. A recent study employed siRNA techniques to show that ROCK1 and ROCK2 actually have separate and opposing effects on keratinocyte differentiation [22]. Lock *et al*. show that knockdown of ROCK2 inhibits differentiation whereas knockdown of ROCK1 promotes keratinocyte differentiation, at least in HaCaT and SCC12F keratinocytes. Another recent study shows that NOTCH1, a key inducer of differentiation, promotes differentiation by activating the ROCK pathway [23]. We believe that inhibition of differentiation and stratification is one of the most important factors responsible for long-term keratinocyte proliferation mediated by ROCK inhibition.

The addition of Y-27632 to the Rheinwald-Green co-culture system results in indefinite keratinocyte proliferation [1]. Suprynowicz and colleagues recently report that cells cultured using our method represent a stem-like state of adult epithelial cells [24]. α6 and β1 integrin, ΔNp63α, CD44 and hTERT are increased and the NOTCH signaling pathway is decreased at the protein level in cells grown in the Rheinwald-Green co-culture system supplemented with Y-27632, when compared to cells grown in commercially available serum free medium [24]. However, as shown previously [20], much of this effect is likely due to the Rheinwald Green culture system and not simply to the inhibition of the ROCK pathway. Here we compare the single variable of ROCK inhibition and do not observe greatly significantly changed levels of these markers at the transcriptional level, although there are small increases in ΔNp63α and TAp63α levels and small decreases in NOTCH2/3. This is consistent with other studies that detect only minor differences in the ROCK transcriptome [25] and indicates that Rho kinases most likely primarily target the phosphoproteome and not the transcriptome. We believe that the differences we observe in the transcriptional profile of HEKs are more likely due to the effect rather than the cause of ROCK inhibition. Other studies also indicate that ROCK inhibitors most likely act at the step of signal transduction, rather than of transcriptional regulation [23].

Our study also shows that several different ROCK inhibitors can promote long-term growth of keratinocytes. No chemical specific for only ROCK1 or ROCK2 kinase is yet commercially available. However, there are many ROCK inhibitors that only affect other kinase pathways at much higher concentrations. We show here that three additional ROCK inhibitors can promote long-term proliferation of keratinocytes. One of these inhibitors, fasudil, is already licensed in Japan and Europe for the treatment of vasospasm after cerebral haemorrhage and is currently undergoing clinical trials for several different cardiovascular conditions, showing that it is safe to use for clinical purposes. Furthermore, small molecules are showing great promise in reprogramming and manipulating the state of stem cells [26]. They have great advantages over genetic manipulation and, as shown here, the effects are often reversible.

We also show that the pro-proliferation and anti-differentiation effect of Y-27632 on keratinocytes is almost immediate. This is in contrast to the notion that a few rare, highly proliferative stem-like cells are being selected for continued passage. With the addition of Y-27632, we observe no obvious effect on keratinocyte migration or ability to form colonies. However, in the absence of the ROCK inhibitor the confluent cells in the centre of the colonies have a propensity to differentiate and stratify. In contrast, cells cultured in Y-27632 proliferate throughout the colony and do not differentiate or stratify, even when tightly packed. Keratinocytes are clonogenic and form several types of colonies in the Rheinwald-Green culture system: holoclones are composed of stem-like cells, which have the greatest proliferative capacity; meroclones are also progenitor cells but are more limited in their ability to generate daughter cells; and, finally, paraclones have the potential to undergo <15 population doublings [27]. Nanba *et al*. show that Y-27632 can inhibit clonal conversion by preventing the formation of paraclones [28], though it cannot revert to a paraclone. Thus, Y27632 maintains the proliferative capacity of progenitor clones and prevents clonal conversion into transit amplifying clones with limited proliferative capacity.

In summary, the addition of a ROCK inhibitor to the Rheinwald-Green keratinocyte method (in which keratinocytes are co-cultured with mitotically inactive fibroblasts) promotes cellular proliferation by inhibition of differentiation and stratification and prevention of clonal conversion. However, this process is reversible and resumption of ROCK activity allows cells to differentiate normally. This method has already proven to be very helpful and valuable for isolating viable, proliferative cells from tiny amounts of tissue [3,5] and provides abundant material for testing therapeutics [4] and for detailed research studies.

## Conclusions

We have previously shown that the addition of a ROCK inhibitor to the Rheinwald-Green keratinocyte culture system induces indefinite cellular proliferation [1]. We show here that this induced proliferation is conditional and reversible, can be extended to animal keratinocytes, and can be mimicked by different ROCK inhibitors. Furthermore, the effect of ROCK inhibition is immediate and involves a dramatic inhibition of differentiation. This simple treatment has far-reaching benefits for personalized regenerative medicine, diagnosis and therapies.

## Abbreviations

DMEM: Dulbecco’s modified Eagle’s medium; EDC: Epidermal differentiation complex; EGF: Epidermal growth factor; FBS: Foetal bovine serum; GO: Gene ontology; HFK: Human foreskin keratinocyte; IC50: Half maximal inhibitory concentration; PKC: Protein kinase C; ROCK: Rho kinase.

## Competing interests

McBride and Chapman are inventors of US Patent 8637310: Use of ROCK Inhibitor, Y-27632, to Sustain Primary Human Keratinocytes in a Proliferative State. Other authors have no competing interests.

## Authors’ contributions

SC and AAM carried out keratinocyte growth studies. MKJ prepared RNA samples for microarray analysis, while KS assisted with this analysis. DM obtained and provided human epidermal keratinocytes. AAM designed the study, analysed the data and drafted the manuscript. All authors read, revrec and approved the final manuscript.

## Supplementary Material

Additional file 1: Table S1 Select datasets from microarray data. **A**, **B**. Microarray data were sorted on “Adjusted *P*-Value for Diff of treatment = (N3 + Y)-(N3)” and all genes with *P* <0.05 are listed in the Y-27632 up-regulated or Y-27632 down-regulated sheet. **C**. Differentially expressed genes identified using the R software package Limma are shown in the Limma differentially expressed genes tab. **D**. Genes previously implicated as epidermal stem cell markers, senescence associated genes or pluripotency-associated genes are listed with their corresponding expression data. Click here for file

Additional file 2: Table S2 Adult human keratinocytes were cultured for several passes in the presence or absence of 10 μM Y-27632. RNA was isolated at passage 3 and passage 4 and was analysed for gene expression by microarray analysis. The major gene ontology (GO) categories that are up-regulated or down-regulated with significance are shown. The complete dataset can be found at GEO GSE52515. Click here for file

Additional file 3: Movie S1 Foreskin keratinocytes cultured for six days in the presence or absence of Y-27632. The movie covers the time period 0 days 13 hours to 4 days 13 hours (see Figure 5) after plating and after the addition of 10 μM Y-27632. Y-27632 was added to the cells for the first time at T = -13 hours with respect to the movie time stamp. Click here for file
