# Supplementary material for: The effect of Rho kinase inhibition on long-term keratinocyte proliferation is rapid and conditional
Source: Stem Cell Res Ther. 2014 Apr 28;5(2):60. doi: 10.1186/scrt449 (PMC4055106; doi:10.1186/scrt449)
Supplement: Additional file 2: Table S2 — Adult human keratinocytes were cultured for several passes in the presence or absence of 10 μM Y-27632. RNA was isolated at passage 3 and passage 4 and was analysed for gene expression by microarray analysis. The major gene ontology (GO) categories that are up-regulated or down-regulated with significance are shown. The complete dataset can be found at GEO GSE52515. [file scrt449-S2.pdf]

The log ratio of the difference in the level of expression of each gene between the mean of the treatment groups (cells cultured in the presence of Y-27632 – level of expression in cells cultured in the absence of Y-27632) was calculated and genes ranked by differential expression. Ranked gene lists were submitted to GOrilla (http://cbl-gorilla.cs.technion.ac.il/) for gene ontology analysis. Shown are GO terms with P values <10E-9. P-value is the enrichment p-value computed according to the mHG or HG model. This p-value is not corrected for multiple testing of 11202 GO terms. FDR q-value is the correction of the p-value for multiple testing using the Benjamini and Hochberg (1995) method. Namely, for the ith term (ranked according to p-value) the FDR q-value is (p-value * number of GO terms) / i. Enrichment = (b/n) / (B/N). N, B, n, b are defined as follows: N - is the total number of genes; B - is the total number of genes associated with a specific GO term; n - is the number of genes at the top of the ranked list; b - is the number of genes in the intersection.

Downregulated by Y-27632 treatment				
GO term	Description	P-value	FDR q-value	Enrichment (N, B, n, b)
GO:0031424	keratinization	5.33E-30	5.97E-26	17.96 (13906,37,586,28)
GO:0009913	epidermal cell differentiation	1.58E-16	8.83E-13	18.49 (13906,72,188,18)
GO:0030216	keratinocyte differentiation	4.75E-16	1.77E-12	21.92 (13906,54,188,16)
GO:0008544	epidermis development	5.97E-16	1.67E-12	10.95 (13906,98,298,23)
GO:0030855	epithelial cell differentiation	5.32E-14	1.19E-10	8.35 (13906,205,203,25)
GO:0018149	peptide cross-linking	3.37E-11	6.29E-08	9.76 (13906,21,950,14)
GO:0009888	tissue development	2.29E-10	3.67E-07	3.01 (13906,431,493,46)
GO:0006629	lipid metabolic process	6.21E-10	8.69E-07	1.68 (13906,851,1467,151)

Upregulated by Y-27632 treatment				
GO term	Description	P-value	FDR q-value	Enrichment (N, B, n, b)
GO:0022402	cell cycle process	2.14E-49	2.40E-45	3.11 (13906,916,992,203)
GO:0006259	DNA metabolic process	8.95E-42	5.01E-38	2.96 (13906,620,1387,183)
GO:0000278	mitotic cell cycle	3.09E-40	1.15E-36	3.59 (13906,376,1379,134)
GO:0006139	nucleobase-containing compound metabolic process	3.39E-33	9.49E-30	1.59 (13906,3441,1362,537)
GO:0006396	RNA processing	3.53E-33	7.90E-30	2.61 (13906,600,1591,179)
GO:0046483	heterocycle metabolic process	1.38E-32	2.57E-29	1.58 (13906,3575,1317,536)
GO:0007049	cell cycle	1.67E-32	2.67E-29	2.81 (13906,568,1379,158)
GO:0090304	nucleic acid metabolic process	1.28E-31	1.80E-28	1.63 (13906,3031,1362,485)
GO:0006725	cellular aromatic compound metabolic process	3.76E-31	4.68E-28	1.57 (13906,3578,1317,532)
GO:0034641	cellular nitrogen compound metabolic process	2.56E-30	2.86E-27	1.54 (13906,3749,1317,548)
GO:1901360	organic cyclic compound metabolic process	3.00E-30	3.05E-27	1.54 (13906,3751,1317,548)
GO:0016071	mRNA metabolic process	3.90E-28	3.64E-25	2.54 (13906,558,1578,161)
GO:0051301	cell division	8.56E-27	7.38E-24	3.95 (13906,314,954,85)
GO:0044260	cellular macromolecule metabolic process	1.12E-26	9.00E-24	1.38 (13906,4804,1605,763)
GO:0006807	nitrogen compound metabolic process	1.70E-25	1.27E-22	1.47 (13906,4065,1317,566)
GO:0010467	gene expression	8.44E-25	5.91E-22	2.32 (13906,645,1589,171)
GO:0006281	DNA repair	8.97E-25	5.91E-22	3.03 (13906,357,1387,108)
GO:0044237	cellular metabolic process	1.43E-24	8.89E-22	1.28 (13906,6290,1613,937)
GO:0006260	DNA replication	3.13E-23	1.84E-20	4.07 (13906,165,1387,67)
GO:0008380	RNA splicing	3.13E-23	1.75E-20	3.15 (13906,287,1462,95)
GO:0000377	RNA splicing, via transesterification reactions with bulged adenosine as nucleophile	1.11E-22	5.93E-20	4.01 (13906,171,1358,67)
GO:0000398	mRNA splicing, via spliceosome	1.11E-22	5.66E-20	4.01 (13906,171,1358,67)
GO:0000375	RNA splicing, via transesterification reactions	1.97E-22	9.59E-20	3.93 (13906,177,1358,68)
GO:0071704	organic substance metabolic process	4.48E-22	2.09E-19	1.29 (13906,6583,1317,804)
GO:0008152	metabolic process	1.10E-21	4.91E-19	1.25 (13906,6865,1613,992)
GO:0006974	response to DNA damage stimulus	1.53E-21	6.59E-19	2.46 (13906,554,1387,136)
GO:0048285	organelle fission	2.16E-21	8.98E-19	3.97 (13906,246,969,68)
GO:0044238	primary metabolic process	2.47E-21	9.87E-19	1.29 (13906,6380,1317,782)
GO:0043170	macromolecule metabolic process	2.72E-21	1.05E-18	1.34 (13906,5261,1411,714)
GO:0006397	mRNA processing	3.58E-21	1.34E-18	2.89 (13906,358,1358,101)
GO:0000280	nuclear division	2.58E-20	9.31E-18	4.05 (13906,223,969,63)
GO:0007067	mitosis	2.58E-20	9.02E-18	4.05 (13906,223,969,63)
GO:0071840	cellular component organization or biogenesis	7.19E-20	2.44E-17	1.52 (13906,3090,1249,423)
GO:0006996	organelle organization	1.11E-19	3.66E-17	1.75 (13906,1605,1390,280)
GO:0044772	mitotic cell cycle phase transition	2.81E-19	9.00E-17	3.60 (13906,253,1053,69)
GO:0044770	cell cycle phase transition	2.81E-19	8.75E-17	3.60 (13906,253,1053,69)
GO:0000082	G1/S transition of mitotic cell cycle	1.15E-18	3.47E-16	4.65 (13906,144,1018,49)
GO:0016043	cellular component organization	2.09E-17	6.15E-15	1.50 (13906,3060,1249,411)
GO:0006310	DNA recombination	1.55E-16	4.46E-14	3.55 (13906,164,1387,58)
GO:0050658	RNA transport	2.11E-16	5.91E-14	3.72 (13906,122,1593,52)
GO:0050657	nucleic acid transport	2.11E-16	5.77E-14	3.72 (13906,122,1593,52)
GO:0051236	establishment of RNA localization	2.11E-16	5.63E-14	3.72 (13906,122,1593,52)
GO:0043933	macromolecular complex subunit organization	2.17E-16	5.65E-14	1.85 (13906,1059,1449,204)
GO:0016072	rRNA metabolic process	3.09E-16	7.87E-14	3.80 (13906,115,1591,50)
GO:0033554	cellular response to stress	3.90E-16	9.72E-14	1.83 (13906,956,1618,204)
GO:0006364	rRNA processing	7.37E-16	1.79E-13	3.85 (13906,109,1591,48)
GO:0015931	nucleobase-containing compound transport	1.48E-15	3.52E-13	3.41 (13906,142,1610,56)
GO:0006271	DNA strand elongation involved in DNA replication	2.16E-15	5.04E-13	7.46 (13906,33,1300,23)
GO:0010564	regulation of cell cycle process	3.12E-15	7.13E-13	2.63 (13906,332,1386,87)
GO:0022616	DNA strand elongation	1.72E-14	3.85E-12	7.03 (13906,35,1300,23)
GO:0051276	chromosome organization	4.72E-14	1.04E-11	2.02 (13906,591,1618,139)
GO:0051726	regulation of cell cycle	4.85E-14	1.05E-11	4.54 (13906,621,207,42)
GO:0034622	cellular macromolecular complex assembly	6.35E-14	1.34E-11	2.44 (13906,399,1329,93)
GO:0034660	ncRNA metabolic process	9.80E-14	2.03E-11	2.53 (13906,287,1591,83)
GO:0016070	RNA metabolic process	1.25E-13	2.55E-11	1.40 (13906,2605,1593,417)
GO:0051028	mRNA transport	1.33E-13	2.66E-11	3.56 (13906,108,1593,44)
GO:0034470	ncRNA processing	2.20E-13	4.32E-11	2.72 (13906,206,1591,64)
GO:0051168	nuclear export	9.42E-13	1.82E-10	3.82 (13906,94,1472,38)
GO:0009987	cellular process	1.23E-12	2.33E-10	1.13 (13906,10051,1272,1040)
GO:0044265	cellular macromolecule catabolic process	1.39E-12	2.60E-10	1.93 (13906,581,1561,126)
GO:0016032	viral process	1.98E-12	3.63E-10	1.93 (13906,563,1584,124)
GO:0044403	symbiosis, encompassing mutualism through parasitism	1.98E-12	3.58E-10	1.93 (13906,563,1584,124)
GO:0044764	multi-organism cellular process	2.65E-12	4.71E-10	1.93 (13906,565,1584,124)
GO:0006412	translation	6.31E-12	1.10E-09	2.45 (13906,250,1566,69)
GO:0006405	RNA export from nucleus	7.54E-12	1.30E-09	4.56 (13906,68,1301,29)
GO:0065003	macromolecular complex assembly	2.13E-11	3.62E-09	2.35 (13906,808,564,77)
GO:0022411	cellular component disassembly	2.27E-11	3.80E-09	2.32 (13906,279,1565,73)
GO:0071822	protein complex subunit organization	2.43E-11	4.00E-09	1.96 (13906,825,982,114)
GO:0007051	spindle organization	4.57E-11	7.42E-09	5.13 (13906,69,982,25)
GO:0007077	mitotic nuclear envelope disassembly	8.37E-11	1.34E-08	5.34 (13906,36,1519,21)
GO:0007059	chromosome segregation	9.79E-11	1.54E-08	3.82 (13906,82,1421,32)
GO:0007017	microtubule-based process	1.02E-10	1.59E-08	3.41 (13906,333,490,40)
GO:0006401	RNA catabolic process	1.81E-10	2.78E-08	2.51 (13906,202,1561,57)
GO:0044419	interspecies interaction between organisms	2.15E-10	3.25E-08	1.81 (13906,607,1584,125)
GO:0030397	membrane disassembly	3.31E-10	4.94E-08	5.06 (13906,38,1519,21)
GO:0051081	nuclear envelope disassembly	3.31E-10	4.88E-08	5.06 (13906,38,1519,21)
GO:0008150	biological_process	5.03E-10	7.32E-08	1.07 (13906,11791,1317,1193)
GO:0000075	cell cycle checkpoint	8.01E-10	1.15E-07	2.95 (13906,141,1370,41)
